# The Impact of Pancreatic Beta Cell Heterogeneity on Type 1 Diabetes Pathogenesis

**DOI:** 10.1007/s11892-018-1085-2

**Published:** 2018-09-24

**Authors:** Richard K. P. Benninger, Craig Dorrell, David J. Hodson, Guy A. Rutter

**Affiliations:** 1 Department of Bioengineering, University of Colorado Anschutz Medical Campus, Aurora, CO 80045 USA; 2 Barbara Davis Center for Childhood Diabetes, University of Colorado Anschutz Medical Campus, Aurora, CO 80045 USA; 3 Oregon Stem Cell Center, Papé Family Pediatric Research Institute, Department of Pediatrics, Oregon Health and Science University, 3181 SW Sam Jackson Park Road, Portland, OR 97239 USA; 4 Institute of Metabolism and Systems Research (IMSR), University of Birmingham, Birmingham, B15 2TT UK; 5 Centre for Endocrinology, Diabetes and Metabolism, Birmingham Health Partners, Birmingham, B15 2TH UK; 6 COMPARE, University of Birmingham and University of Nottingham Midlands, Nottingham, UK; 7 Section of Cell Biology and Functional Genomics, Department of Medicine, Imperial College London, London, W12 0NN UK

**Keywords:** Beta cell, Type 1 diabetes, Insulin, Heterogeneity, Transcriptomics, Imaging

## Abstract

**Purpose of Review:**

To discuss advances in our understanding of beta-cell heterogeneity and the ramifications of this for type 1 diabetes (T1D) and its therapy.

**Recent Findings:**

A number of studies have challenged the long-standing dogma that the majority of beta cells are eliminated in T1D. As many as 80% are present in some T1D subjects. Why don’t these cells function properly to release insulin in response to high glucose? Other findings deploying single-cell “omics” to study both healthy and diseased cells—from patients with both T1D and type 2 diabetes (T2D)—have revealed cell subpopulations and heterogeneity at the transcriptomic/protein level between individual cells. Finally, our own and others’ findings have demonstrated the importance of functional beta-cell subpopulations for insulin secretion.

**Summary:**

Heterogeneity may endow beta cells with molecular features that predispose them to failure/death during T1D.

## Introduction

The incidence rate of type 1 diabetes (T1D) is ~20 per 100,000 individuals under 15 years in the USA and UK, with 13,000 new cases diagnosed each year [1]. Classically, T1D has been thought to involve the progressive, and ultimately near-complete (> 90%), destruction of beta cells within the pancreatic islet [2]. The selective loss of beta cells results from immune attack mediated by CD4+ and CD8+ T cells recruited by islet-derived auto-antigens [3, 4], notably insulin, GAD65, IA-2, ZnT8 [5], Chromogranin A (ChgA) [6], and IGRP (G6PC2) [7], as well as hybrid peptides formed between insulin and ChgA or IAPP [8]. Correspondingly, islet auto-antigen load is predictive of seroconversion to T1D [9].

Auto-antigens are presented at the beta-cell surface by class I human leukocyte antigen (HLA; also called major histocompatibility complexes, MHC) and are presented by antigen presenting cells by class II HLA molecules. Emphasizing the importance of antigen presentation in disease etiology, the genetic variants most strongly associated with T1D risk are HLA class II genes, with HLA class I genes also contributing. In addition, genetic variants associated with T1D risk include those encoding T cell activity and antigen recognition (e.g., PTPN2 [10], CTLA4 [11]), and cytokine signaling (e.g., IL2, IL2RA, IL10). Nonetheless, the extent to which this reflects failures in the immune system versus alterations at the level of the beta cell is not fully resolved. For example, PTPN2 plays an important role in beta-cell responses to inflammatory cytokines, including apoptosis [10].

Although the classical conception of T1D involves the essentially complete loss of beta cells in T1D, more recent studies [12–14] have demonstrated that a significant proportion of T1D patients retain detectable levels of circulating C-peptide, indicating at least partially preserved insulin secretion and (partly) functional beta cells. Correspondingly, at the histological level, patients diagnosed with T1D at < 7 years of age display a “classical” picture of > 90% loss of insulin-containing islets, whereas those diagnosed later (> 13 years) retain a substantial fraction (20–80%) [15]. It is possible that less-functional cells among a heterogeneous population selectively survive, as has been suggested by functional heterogeneity among beta cells in T2D [16, 17]. At present, however, the mechanisms through which the extant beta-cell population is rendered sub-functional in T1D have remained obscure.

Consideration of how healthy pancreatic beta cells respond to acute challenge with elevated glucose concentrations is relevant here. High glucose levels prompt accelerated uptake and metabolism of the sugar, chiefly controlled by flux through the low-affinity glucose-phosphorylating enzyme glucokinase (hexokinase IV) [18]. Enhanced generation of ATP by mitochondria [19], and the closure of ATP-sensitive K^+^ channels (K_ATP_) [20], then leads to Ca^2+^ influx through voltage-gated Ca^2+^ channels [21]. Other pathways, including mitochondrial GTP synthesis [22], as well as the mitochondrial export of metabolites including citrate [23], isocitrate [24] or glutamate [25], may further potentiate the effects of increased Ca^2+^. Importantly, glucose-stimulated insulin secretion is pulsatile, and likely to be driven by low- (5–15 min) and high- (1–5 min) frequency oscillations in intracellular free Ca^2+^ [26].

Studies in humans [27] reveal relatively modest effects on serum fasting glucose and glucose tolerance from the surgical removal of 50% of the pancreas, while in non-human primates [28], hemi-pancreatectomy had more marked effects. These findings make it unlikely that frank diabetes in patients with > 50% of their beta cells remaining can be attributed to beta-cell loss alone (note, however, that there are wide inter-individual differences in beta-cell mass between non-diabetic individuals [29] making the definition of “normal” beta-cell mass challenging).

Why are the remaining beta cells from these patients unable to produce sufficient hormone to control blood glucose levels? Whereas defects in the mitochondrial oxidative metabolism have been described in islets from type 2 diabetes (T2D) patients [30], and likely contribute to impaired insulin secretion in that disease, their role, if any, in T1D islets is uncertain. Of note, the existing genetic data argue for distinct etiologies for T1D and T2D, with relatively little overlap between the genome-wide association *loci* identified in studies for T2D [31] and T1D [32]. Indeed, only a single locus (*GLIS3*), of the hundreds identified for each disease, overlaps in T1D and T2D. However, recent studies [33] have increased the number of T2D loci (~420). The study of these may reveal greater overlap with the 60 variants associated with T1D, and thus, potentially, some shared disease mechanisms.

Islet function is impaired prior to the onset of diabetes in animal models of T1D [34, 35]. In human, in vivo measurements show signs of beta-cell dysfunction up to 5 years prior to diabetes onset [36•]. These findings suggest that beta cells become defective before they are killed outright. Correspondingly, insults that pertain to T1D, e.g., the release from inflammatory cells of sub-lethal cytotoxic cytokines such as interferon γ (IFNγ), interleukin-1β (IL-1β), and tumor necrosis factor-α (TNF-α), impair beta cell metabolism and the usual increases in cytosolic ATP/ADP ratio following nutrient stimulation. These effects are observed both in vivo in non-obese diabetic (NOD) mice [37], and in vitro [38] in isolated islets, and are thus likely to compromise glucose-stimulated insulin secretion.

In this report, we discuss the notion that beta cell heterogeneity and the existence of functional beta cell subpopulations [39, 40] may be relevant to the normal control of insulin secretion and may become defective in the context of T1D. Furthermore, we raise the possibility that the preservation of heterogeneity may provide a new preventative or therapeutic strategy in some settings.

## Single Cell and Subpopulation Analyses of Beta Cell Heterogeneity

Although the concept of functional and phenotypic beta cell heterogeneity is not new, advances in single-cell analysis have contributed to an explosion of information on this topic in the last 3 years. Nearly all of these examined either mouse cells or human cells from healthy or T2D donor islets, in part due to the limited availability of T1D islets. Nevertheless, “baseline” heterogeneity and a pathological heterogeneity should have implications for type 1 diabetes as well.

The Kubicek lab first reported the application of single-cell transcriptomics to human islet cells. In Li et al. [41], they reported the comparison of 64 human pancreatic cells isolated by fluorescence-activated cell sorting (FACS) and processed by Smart-Seq2 for high-throughput sequencing. Although only 12 beta cells were identified, these showed transcriptome heterogeneity for genes such as DLK1 (which has been associated with T1D by GWAS [42]). In another first, the Kaestner lab used CyTOF single-cell mass cytometric analysis to interrogate the levels of 24 different proteins on millions of human islet cells [43]. Heterogeneous levels of several markers were observed in beta cells, including CD9 (see below) and the proliferation marker Ki67, revealing four subpopulations after viSNE analysis (a variant of Stochastic Neighbour Embedding, t-SNE, used to present single-cell data in two dimensions [44]).

Several groups have now performed large-scale single-cell transcriptome analyses of human beta cells [16, 45–49], mouse beta cells [50, 51], or both [52]; those which focus on the changes associated with T2D will be discussed later. Work performed in the laboratory of van Oudenaarden identified three beta cell subpopulations by RaceID clustering and found that some of the strongest genes distinguishing these clusters were involved in ER/oxidative stress [46]. Baron et al. [52] also reported stress marker-related heterogeneity in normal human beta cells, but their principal component analysis resolved two populations. Differential expression of UCN3, a marker of beta cell maturity [53], was also observed. A recent publication from the Gromada group supports the idea that human beta-cell subpopulations are distinguished by different ER stress states and describes differential expression of some of the same genes [49]. In addition, this report links ER stress with proliferative capacity and a reduction of beta cell function including insulin production. Chronic ER stress was also shown to correlate with heterogeneous expression of aging markers including *Igf1r* [54]. Enge et al. [48], in contrast, report age-related increases in transcriptional “noise” within the beta cell transcriptome, but cell subtypes were not detected.

Zeng et al. [50] and Qiu et al. [51] performed single-cell transcriptome analyses of mouse beta cells. In the Zang study, transcriptional heterogeneity at observed and projected time points was compared by arranging trajectories based on transcriptome similarity (“pseudotimelines”). The authors conclude that heterogeneity is persistent and that, consistent with the report above, ROS-induced ER stress promotes proliferation of the associated cell subset [50]. Qiu et al. report a low degree of transcriptome heterogeneity in mature mouse beta cells, but point out that post-transcriptionally specified heterogeneity would not be detected in these studies [51].

**Fig. 1:**
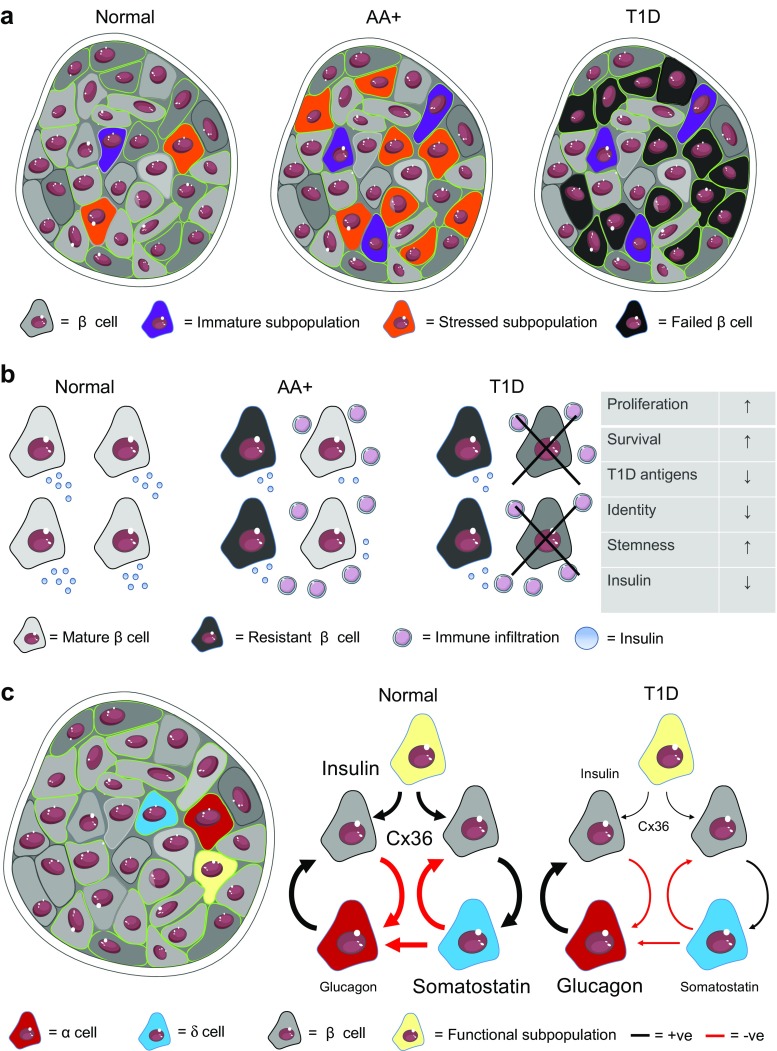
Working interpretation of the role of beta cell heterogeneity in T1D. **a** Beta cells possess molecular heterogeneity giving rise to subpopulations, some of which are functionally competent. Shifts in the proportions of these subpopulations, in particular those with proliferative or ER-stressed phenotypes, may be expected to occur during T1D progression. **b** Beta cell subpopulations that are resistant to immune attack occur in NOD mice, with lowered insulin release, lowered expression of genes for function and metabolism, increased expression of genes for T1D antigens (AA; auto-antigen), but increased markers of proliferation, stemness, and survival. The table shows characteristics of immune attack-resistant cells characterized in [76]. **c** The islet hosts electrical (gap junction; Cx36) and paracrine loops, which give rise to functional beta-cell subpopulations. Failure in intercellular communication has been shown to occur in response to pro-inflammatory conditions [39, 83]. Figures were adapted from Servier Medical Art under a CC-BY3.0 license (https://creativecommons.org/licenses/by/3.0/)

Although these studies do not directly assay or model T1D, elements of the observed heterogeneity are potentially quite relevant. The inflammatory environment to which a beta cell is exposed during insulitis is known to promote proliferation [55], and it seems plausible that subpopulations of normal beta cells that demonstrate proliferative capacity would be those most likely to respond in T1D. In addition, reports of ER stress-related heterogeneity seem relevant to the T1D environment, where ER stress is strongly induced [56]. These single-cell studies may reveal changes in heterogeneity reflecting differential survival of subtypes and/or adaptations to the progressive immune assault on the beta cell pool (Fig. 1a). Of note, the surviving cell population includes few if any proliferating cells [58], perhaps suggesting preferential killing of dividing cells (with some exceptions as broached later). Intriguingly, prior to disease onset, antibody-positive subjects were found to have unaltered beta cell mass (as assessed by insulin positivity) but an increased proinsulin-positive area, perhaps suggestive of (a) increased proliferation prior to immune attack and (b) impaired function or cellular “identity” [59].

## Functional Heterogeneity of Healthy Beta Cells Within the Intact Islet

Pioneering work conducted almost 30 years ago provided the first evidence that beta cells display marked functional heterogeneity, including differences in ion channel conductance, Ca^2+^ fluxes, metabolism, insulin expression/secretion, and proliferation [60–63]. Such heterogeneity may also render beta cells sensitive to insult: the most glucose-sensitive beta cells are also the most susceptible to cytokine-induced stress [64], whereas proliferation is lowest in cells with the highest levels of pro-inflammatory NF-κB signaling [65]. Heterogeneity is further shaped by the islet context, where beta cells are electrically coupled via gap junctions, and are also subjected to modulatory inputs from neighboring cells (e.g., α cells, δ cells) [66–70], together ensuring the coordinated regulation of insulin secretion. The complex signaling interactions afforded by the islet architecture give rise to “functionally competent” metabolically adapted subpopulations that are able to exert disproportionate influence over islet function [39, 40]. Whether this leads to greater robustness of the islet, or actually increases susceptibility, is unclear. Similarly, transcriptionally immature subpopulations have been discovered in the adult islet that display reduced glucose uptake, mitochondrial function, and Ca^2+^ fluxes, but enhanced proliferative capacity [71, 72]. These studies suggest an association between proliferation state and immaturity: indeed c-myc overexpression to force replication induces a neonatal-like beta cell state [73]. Notably, single-cell sequencing approaches applied to dissociated cells are unlikely to fully recapitulate the heterogeneity imparted by interactions at the level of the islet, especially as transcriptional changes can occur rapidly following isolation and sensitivity of even RNASeq is still relatively limited. Moreover, some of the “functionally competent” subpopulations identified so far are more sensitive to stress, possibly due to lowered Pdx1 levels or glucokinase activity [74, 75], and may occupy an apoptotic or necrotic fraction that is discarded following dissociation [39]. Other possibilities include the dependence of functional subpopulations on islet architecture for their phenotype, or changes in stability (original studies only tracked fate over a few hours [39]). Key questions therefore include whether the stresses associated with T1D drive beta cells towards more or less proliferation, or affect differentiation state and identity: it would be of interest to determine in T1D islets the proportion of the four beta-cell subtypes as described recently [77]. The fact that cryopreserved pancreata comprise the bulk of samples from individuals with T1D reinforces the need to deploy approaches that can interrogate beta cells directly in situ in the tissue context with high precision (e.g., laser microdissection followed by RNASeq; STARmap, CLARITY, imaging mass cytometry/multiplexed ion beam imaging, or other highly multiplexed imaging approaches) [78–81].

Implying functional relevance for connections between functionally heterogeneous beta cells, we [66, 70] and others [67] have shown the critical importance of gap junctions, formed by connexin36 (Gjd2), for the normal control of insulin secretion. Importantly, we have shown that aging [82] or conditions mimicking the diabetic milieu, including those relevant to T1D such as elevated cytokine levels [66, 83], impair beta cell to beta cell communication. This is also altered—as measured in isolated islets—as a function of body mass index, consistent with a role for lowered intercellular communication in the metabolic syndrome and prediabetes [66]. Analogous studies are lacking in islets obtained from T1D subjects.

## Changes in Beta Cell Identity and Heterogeneity in T1D and T2D

Several recent reports have emphasized the likely importance of changes in beta-cell identity in T2D at the population (i.e., whole islet) level [84, 85]. The extent to which this may reflect changes in the expression of genes within distinct beta-cell subpopulations, or altered ratios of these subpopulations, in disease is unknown.

A report by Wang et al. [17] was the first to describe single-cell transcriptome analysis of human beta cells from a T1D islet. Unfortunately, the difficulty of obtaining and processing such islets and the scarcity of beta cells within them meant only six cells were analyzed and no conclusions could be reached regarding heterogeneity. However, by comparing beta cells from T2D and healthy donors, they observed many cells that appeared to have de-differentiated, adopting a transcriptome resembling juvenile beta cells. The Sandberg laboratory also compared normal and T2D beta cells, and identified five subpopulations [16]. Among the differentially expressed genes was RBP4, which has been reported to be downregulated in T1D patients [86]. Xin et al. [45] and Lawlor et al. [47] did not detect beta cell subpopulations in their respective T2D vs. healthy islet cell-type comparisons, but the latter reported expression of T1D-associated MEG3 [42] in human beta cells. A renewed interest in the application of single-cell analysis to surviving beta cells in T1D islets should help to determine whether changes in heterogeneity are associated with this disease as well.

How might heterogeneity in the islet contribute to T1D? Pro-inflammatory cytokines associated with islet dysfunction during T1D are known to perturb many facets of beta cell function including metabolic and electrical activity, insulin granule synthesis/content, and gap junction coupling [39, 83, 87]. This could feasibly alter vulnerability to insult as well as shift the balance between the different functional subpopulations. Thus, subpopulations of immature proliferative beta cells with reduced functional capacity are relatively resistant to cell death [71, 76••]. On the other hand, increased beta cell metabolic or electrical activity can increase susceptibility to cell death [88, 89].

In particular, a population of beta cells has been shown to develop during the onset of T1D in NOD mice. These cells have characteristics of immaturity, including reduced insulin granule content and increased expression of genes for proliferation, immune inhibition, and “stemness” [76••]. “Deep phenotyping” of the resistant population showed changes in beta cell identity redolent of those observed in human type 2 diabetes [18] and included the upregulation of beta-cell “disallowed” genes [90], such as Acot7 [91] (Fig. 1b). These findings again point to similar dysfunction in the case of surviving T1D beta cells and the changes observed in T2D. The extent to which these changes are reactive, i.e., the consequence in both instances of hyperglycemia or the inflammatory environment, requires further exploration. Additionally, an interesting recent report [92] demonstrates that expression on beta cells of co-inhibitory receptor-programmed death-ligand 1 (PD-L1) restrains T cell reactivity in both non-obese diabetic (NOD) mice and human T1D patients, suggestive of an attempt by beta cells to escape cell killing. Whether the induction of this gene reflects an intrinsic (i.e., cell autonomous) property of the responding beta cells (and thus which sub-population?), rather than the local nature of T cell exposure, represents an interesting question for the future.

Conversely, metabolically adapted and functionally competent beta cells are more sensitive to the effects of the cytokines IL-1β and interleukin-6 (IL-6) [39], and would be expected to fail early in T1D due to ER stress given their low levels of sarco(endo-)plasmic reticulum Ca^2+^ ATPase-2 (SERCA2). This probably reflects the differing proliferative and glucose uptake capacities of the different subpopulations [64, 71, 73], as well as the sensitivity of electrical coupling to inflammatory cytokines and other insults [66, 83]. In any case, function and proliferation need to be carefully controlled in the normal islet and shifts to either extreme may render beta cells susceptible to failure during T1D. Paracrine pathways may also become perturbed during T1D, leading to apparent loss of heterogeneity. For example, a reduction in beta cell function and/or number would be expected to increase glucagon secretion due to loss of negative feedback. Likewise, changes in α cell or δ cell function and/or number may dysregulate glucagon, somatostatin, and insulin secretion due to the loss of paracrine feedback loops between islet cell subtypes, notably those feedback loops existing between beta cells and delta cells or between alpha cells and delta cells that serve to set the range of insulin and glucagon secretion [68]. From this, it should be clear that functional heterogeneity in the islet may exert a large and dynamic influence on beta cell function during T1D (Fig. 1c). Methods to preserve specific subpopulations or paracrine signals are thus likely to increase the resilience of islets during the early stages of T1D.

## Relevance of Beta Cell Heterogeneity for T1D Pathogenesis and Therapy

Studies of isolated islets from patients with T1D have shown that biphasic insulin secretion can be restored in some individuals following culture in a non-diabetogenic environment [93]. However, it is unlikely that the function of the remaining beta cells can be rescued in vivo, as studies in patients with allografts plus immunosuppression showed no restoration of endogenous insulin release [12]. However, further studies are warranted during the earlier stages of T1D when previous immune attack and destruction may not be so extensive, or following treatments that exert longer term and improved glycemic control (e.g., closed loop pumps) [78]. Many current immunosuppressive regimes, including the use of rapamycin (sirolimus) [94], exert deleterious effects on beta cell function via effects on mitochondrial respiration and as such may not be optimal during islet transplantation. Furthermore, clinical trials are only beginning to demonstrate rescuing beta cell function as a therapeutic strategy (e.g., Verapamil [95•]).

Another consideration is whether beta cell subpopulations known to be susceptible to stress—and which might therefore be preferentially rendered inactive or destroyed in disease—can be protected to preserve islet function for as long as possible during infiltration. While both immune infiltration and beta cell decline have been shown to be highly variable in T1D [96•], functionally competent-sensitive subpopulations may nevertheless succumb early. This is consistent with the rapid decline in hub cells and gap junction coupling following pro-inflammatory cytokine exposure [39, 83]. On the other hand, the emergence of cells resistant to cell death occurs as early as 3–4 weeks in NOD mice when insulitis is very mild [76••]. A better understanding of the role for such subpopulations will be key to developing a clearer picture of their fate during T1D, as well as their cellular and molecular signatures. Along similar lines, can immune attack-resistant beta cell subpopulations be harnessed either early in T1D to confer resistance, or conversely repopulated later in T1D using, e.g., in vivo reprogramming strategies [97]? It will be important to study whether these populations are functional on a background of loss of other beta cell subpopulations due to changes in paracrine and electrical inputs.

Lastly, stem cell-derived beta cells or islets are likely to be transformative in the treatment of T1D following encapsulation to decrease immune attack [98]. Engineering such tissue to possess highly functional and/or immune-resistant beta cell subpopulations could increase graft longevity and performance, especially if such stem cell-derived beta cells prove to be less immunogenic.

## Conclusions

It should be clear that beta cell heterogeneity is critical for normal islet function, and shifts in this parameter may predispose beta cells to metabolic stress. While we recognize that most studies so far have been performed in tissue from healthy or T2D individuals, many molecular features of the identified beta cell subpopulations could render them more susceptible to both immune damage (i.e., ER stress) and loss of insulin secretory capacity (i.e., proliferation) during T1D. Using next-generation approaches applied to isolated islets, or more likely cryo-preserved pancreatic tissue, it will be interesting to see which features of heterogeneity may be preserved (or different) between T1D and T2D.
